# Mediating effect of soluble B-cell activation immune markers on the association between anthropometric and lifestyle factors and lymphoma development

**DOI:** 10.1038/s41598-020-70790-9

**Published:** 2020-08-14

**Authors:** Fatemeh Saberi Hosnijeh, Pieter M. Kolijn, Delphine Casabonne, Alexandra Nieters, Marta Solans, Sabine Naudin, Pietro Ferrari, James D. Mckay, Elisabete Weiderpass, Vittorio Perduca, Caroline Besson, Francesca Romana Mancini, Giovanna Masala, Vittorio Krogh, Fulvio Ricceri, José M. Huerta, Dafina Petrova, Núria Sala, Antonia Trichopoulou, Anna Karakatsani, Carlo La Vecchia, Rudolf Kaaks, Federico Canzian, Dagfinn Aune, Heiner Boeing, Matthias B. Schulze, Aurora Perez-Cornago, Anton W. Langerak, Vincent H. J. van der Velden, Roel Vermeulen

**Affiliations:** 1 Department of Immunology, Laboratory Medical Immunology, Erasmus MC, University Medical Center, Rotterdam, The Netherlands; 2 Division of Environmental Epidemiology, Institute for Risk Assessment Sciences, Utrecht University, P.O. Box 80178, 3508 TD, Utrecht, The Netherlands; 3 Centro de Investigación Biomédica en Red de Epidemiología y. Salud Pública, M.P. (CIBERESP), Madrid, Spain; 4 Unit of Infections and Cancer, Cancer Epidemiology Research PRogramme, Catalan Institute of Oncology, IDIBELL, L’Hospitalet de Llobregat, Spain; 5 Faculty of Medicine and Medical Center, Institute for Immunodeficiency, University of Freiburg, Freiburg, Germany; 6 Research Group on Statistics, Econometrics and Health (GRECS), University of Girona, Girona, Spain; 7 Nutritional Methodology and Biostatistics Group, International Agency for Research on Cancer, World Health Organization, Lyon, France; 8 Section of Genetics, International Agency for Research on Cancer, Lyon, France; 9 International Agency for Research on Cancer– World Health Organization, Lyon, France; 10 CNRS, MAP5 UMR 8145, Université de Paris, 75006 Paris, France; 11 CESP, Fac. de Médecine - Univ. Paris-Sud, Fac de Médecine - UVSQ, INSERM, Université Paris Saclay, 94805 Villejuif, France; 12 Gustave Roussy, 94805 Villejuif, France; 13 Department of Hematology and Oncology, Hospital of Versailles, Le Chesnay, France; 14 Cancer Risk Factors and Life-Style Epidemiology Unit, Institute for Cancer Research, Prevention and Clinical Network - ISPRO, Florence, Italy; 15 Epidemiology and Prevention Unit, Fondazione IRCCS Istituto Nazionale dei Tumori di Milano, Milan, Italy; 16 Department of Clinical and Biological Sciences, University of Turin, Turin, Italy; 17 Unit of Epidemiology, Regional Health Service ASL, Turin, Italy; 18 Department of Epidemiology, Murcia Regional Health Council, IMIB-Arrixaca, Murcia, Spain; 19 CIBER of Epidemiology and Public Health (CIBERESP), Madrid, Spain; 20 Andalusian School of Public Health (EASP), Granada, Spain; 21 Instituto de Investigación Biosanitaria de Granada (Ibs.GRANADA), Universidad de Granada, Granada, Spain; 22 Unit of Nutrition, Environment and Cancer, Cancer Epidemiology Research Program and Translational Research Laboratory, Catalan Institute of Oncology (ICO), Biomedical Research Institute (IDIBELL), Barcelona, Spain; 23 Hellenic Health Foundation, Athens, Greece; 24 Pulmonary Medicine Department, School of Medicine, National and Kapodistrian University of Athens, “ATTIKON” University Hospital, Haidari, Greece; 25 Department of Clinical Sciences and Community Health Università Degli Studi di Milano, 20133 Milan, Italy; 26 Division of Cancer Epidemiology, German Cancer Research Center (DKFZ), Heidelberg, Germany; 27 Research Group Genomic Epidemiology, German Cancer Research Center (DKFZ), Heidelberg, Germany; 28 Department of Epidemiology and Biostatistics, School of Public Health, Imperial College London, London, UK; 29 Department of Nutrition, Bjørknes University College, Oslo, Norway; 30 Department of Endocrinology, Morbid Obesity and Preventive Medicine, Oslo University Hospital, Oslo, Norway; 31 Department of Epidemiology, German Institute of Human Nutrition Potsdam-Rehbruecke, Nuthetal, Germany; 32 Department of Molecular Epidemiology, German Institute of Human Nutrition Potsdam-Rehbruecke, Nuthetal, Germany; 33 Institute of Nutritional Sciences, University of Potsdam, Nuthetal, Germany; 34 Cancer Epidemiology Unit, Nuffield Department of Population Health, University of Oxford, Oxford, UK; 35 Julius Center for Health Sciences and Primary Care, University Medical Center Utrecht, Utrecht, The Netherlands; 36 Department of Epidemiology and Biostatistics, MRC-PHE Centre for Environment and Health, Imperial College London, London, UK

**Keywords:** Risk factors, Biomarkers, Predictive markers, Cancer epidemiology, Haematological cancer, Lymphoma

## Abstract

Sustained B-cell activation is an important mechanism contributing to B-cell lymphoma (BCL). We aimed to validate four previously reported B-cell activation markers predictive of BCL risk (sCD23, sCD27, sCD30, and CXCL13) and to examine their possible mediating effects on the association between anthropometric and lifestyle factors and major BCL subtypes. Pre-diagnostic serum levels were measured for 517 BCL cases and 525 controls in a nested case–control study. The odds ratios of BCL were 6.2 in the highest versus lowest quartile for sCD23, 2.6 for sCD30, 4.2 for sCD27, and 2.6 for CXCL13. Higher levels of all markers were associated with increased risk of chronic lymphocytic leukemia (CLL), follicular lymphoma (FL), and diffuse large B-cell lymphoma (DLBCL). Following mutual adjustment for the other immune markers, sCD23 remained associated with all subtypes and CXCL13 with FL and DLBCL. The associations of sCD23 with CLL and DLBCL and CXCL13 with DLBCL persisted among cases sampled > 9 years before diagnosis. sCD23 showed a good predictive ability (area under the curve = 0.80) for CLL, in particular among older, male participants. sCD23 and CXCL13 showed a mediating effect between body mass index (positive) and DLBCL risk, while CXCL13 contributed to the association between physical activity (inverse) and DLBCL. Our data suggest a role of B-cell activation in BCL development and a mediating role of the immune system for lifestyle factors.

## Introduction

B-cell lymphomas (BCL) are an etiologically, clinically, and histologically heterogeneous group of malignant diseases of B lymphocytes. Immunodeficiency and autoimmunity are strong B-cell lymphoma risk factors. Chronic B-cell activation is suspected to be an important mechanism contributing to the accumulation of genetic errors that can lead to lymphomagenesis^1^. Increased serum/plasma levels of molecules involved in B-cell activation, among which soluble (s)CD23, sCD27, sCD30, sCD44, and CXCL13, have been associated with the development of acquired immune deficiency syndrome (AIDS)-related BCL^2–7^. Recently, studies within general population cohorts incorporating serologic measurements of cytokines, chemokines, and other immune markers have provided important evidence supporting a role for subtle immunologic effects in lymphomagenesis even among non-immunocompromised individuals^8–12^. Elevated serum levels of sCD23, sCD27, sCD30, and CXCL13 have subsequently been shown to be associated with BCL development in immunocompetent individuals^8–12^.

The risk of BCL has been associated with lifestyle, viral, and environmental factors^13,14^. A large study from the International Lymphoma Epidemiology Consortium (InterLymph) showed several risk/protective factors for Non-Hodgkin lymphoma subtypes^13^. These included a family history of hematologic malignancy, history of autoimmune disease, hepatitis virus C (HCV) infection, body mass index (BMI), height, smoking, and occupation, which were all associated with increased risk of NHL and/or one of its subtypes (CLL, FL, or DLBCL), while alcohol intake (≥ 1 drink per month), better socio-economic position (SEP), history of atopic disease, and recreational sun exposure were linked with reduced risks. Given the central role of the immune system in lymphomagenesis, most risk factors may influence BCL risk through modulation of the immune function^15^. Indeed, diet and obesity have an important influence on the immune system as immune functions are sensitive to both under- and over-nutrition^16^. Obesity promotes increased production of cytokines and leptin^16^, and the latter, in turn, has been shown to enhance B-cell survival^17^.

Infection with hepatitis virus B (HBV) and HCV correlate with high sCD30^18–20^ and sCD23^21,22^ serum levels. In a study including HCV-infected patients, with and without BCL, a signature involving sCD27, sIL-2Rα, gamma globulins, and complement factor 4 was associated with the presence of overt BCL in HCV-infected patients^23^.

Moreover, exposure to environmental factors suspected to be acting as lymphomagens (i.e. trichloroethylene and 2,3,7,8-tetrachlorodibenzo-p-dioxin) altered sCD27 and sCD30 plasma levels^24,25^. Studies have shown that alcohol consumption may be associated with a decreased risk of BCL^13^. Alcohol is associated with dysregulation of cytokines and chemokines, which may mediate alcohol-induced tumor promotion. Alcohol may also affect transcription factors and signaling pathways that regulate the expression/function of cytokines and chemokines^26^. Alcohol abuse impairs both the number and function of B cells. Chronic alcohol consumption reduces B-cell numbers, decreases antigen-specific antibody responses, increases the production of auto-antibodies, and interferes with B-cell development and maturation. Moreover, alcohol’s impact on T and B cells increases the risk of infections (e.g., pneumonia, HIV infection, hepatitis C virus infection, and tuberculosis)^27^. However, to date, the direct mediating effect of immune markers on the association between these factors and BCL risk has not been investigated in a prospective setting.

In our previous study among participants of two prospective cohorts (32 BCL cases overlap with cases included in the present study), which also included a meta-analysis of published data on sCD27 and sCD30, we reported a highly consistent association between these markers and increased risk of BCL subtypes^12^. Pre-diagnostic sCD23 was a strong predictive marker (area under the curve (AUC) = 0.88) for diagnosis of CLL (179 CLL cases overlap with cases included in the present study)^28^. So far, previous studies have limitations in terms of sample size, in particular for BCL subtypes, limiting their power to detect associations of moderate magnitude.

In the present study, we aimed to extend our previous findings using a much larger population within the European Prospective Investigation into Cancer and Nutrition (EPIC) population to examine the relationship between pre-diagnostically level of the most promising previously reported B-cell activation markers (sCD23, sCD27, sCD30, and CXCL13) and subsequent development of BCL major subtypes, i.e. diffuse large B-cell lymphoma (DLBCL), follicular lymphoma (FL), and chronic lymphocytic leukemia (CLL). Moreover, we aimed at exploring the potential clinical utility of these markers for screening and their possible mediating effects on the association between anthropometric and lifestyle factors and BCL subtypes.

## Results

Table 1 shows the characteristics of the study population. Overall, case and control subjects did not differ with regard to risk factors and covariates. Median time between recruitment (i.e., blood collection) in the study and diagnosis of BCL was 9 years (range, 0.2–19). Blood levels of all B-cell activation markers were significantly higher in cases compared to controls (Table 1). Spearman correlations were calculated between the various immune markers among the total BCL cases, controls, and by lymphoma subtypes, ranging from 0.14 to 0.63 (Supplementary Table 1). We found the lowest correlations between these immune markers among controls, while sCD30 was highly correlated with sCD27 in CLL, and with all other markers in FL. Levels of the markers were positively correlated with age in both cases and controls except for CXCL13 in control subjects (Supplementary Fig. 1). Compared to male participants, female subjects had slightly higher levels of sCD30 and CXCL13 that met the threshold of statistical significance (Supplementary Table 2). There was no significant difference in levels of the markers in different countries except for CXCL13 (Supplementary Table 2).

**Table 1:** General characteristics of B-cell lymphoma cases and controls.

	Matched pairs, n = 1,032			All subjects, n = 1,042^b^		
	Cases, n = 516	Controls, n = 516	*p*	Cases, n = 517	Controls, n = 525	*p*
Age^a^	55.2 (9.11)	55.2 (9.11)	0.99	55.2 (9.09)	55.2 (9.08)	0.92
Female, n (%)	301 (58.3)	301 (58.3)	1.00	301 (58.2)	305 (58.1)	1.00
Height, cm^a^	166.3 (9.09)	165.8 (9.39)	0.35	166.3 (9.09)	165.8 (9.38)	0.37
BMI, kg/m^2a^	26.6 (4.40)	26.3 (3.92)	0.26	26.6 (4.39)	26.3 (3.91)	0.26
**Education, n (%)**			0.40			0.41
None	41 (7.9)	37 (7.2)		41 (7.9)	38 (7.2)	
Primary school	175 (33.9)	159 (30.8)		176 (34)	162 (30.9)	
Technical/professional school	131 (25.4)	121 (23.4)		131 (25.3)	123 (23.4)	
Secondary school	74 (14.3)	92 (17.8)		74 (14.3)	92 (17.5)	
University/college	95 (18.4)	107 (20.7)		95 (18.4)	110 (21)	
**Smoking status, n (%)**			0.98			0.98
Never smoker	238 (46.1)	241 (46.7)		238 (46)	244 (46.5)	
Former smoker	168 (32.6)	167 (32.4)		169 (32.7)	169 (32.2)	
Current smoker	110 (21.3)	108 (20.9)		110 (21.3)	112 (21.3)	
**Alcohol intake, gr/day**^**c**^	5.5 (0–124.2)	4.7 (0–235.2)	0.56	5.5 (0–124.2)	4.7 (0–235.2)	0.67
**Physical activity, n (%)**			0.93			0.96
Inactive	136 (26.4)	135 (26.2)		137 (26.5)	137 (26.1)	
Moderately inactive	159 (30.8)	166 (32.2)		159 (30.8)	168 (32)	
Moderately active	110 (21.3)	112 (21.7)		110 (21.3)	113 (21.5)	
Active	111 (21.5)	103 (20)		111 (21.5)	107 (20.4)	
**Country, n (%)**			1.00			1.00
Italy	100 (19.4)	100 (19.4)		100 (19.3)	104 (19.8)	
Spain	95 (18.4)	95 (18.4)		95 (18.4)	96 (18.3)	
UK	143 (27.7)	143 (27.7)		144 (27.9)	143 (27.2)	
Netherlands	96 (18.6)	96 (18.6)		96 (18.6)	97 (18.5)	
Greece	22 (4.3)	22 (4.3)		22 (4.3)	22 (4.2)	
Germany	60 (11.6)	60 (11.6)		60 (11.6)	63 (12)	
**sCD23, pg/mL**^a^	8.15 (0.66)	7.71 (0.39)	**< 0.0001**	8.15 (0.66)	7.71 (0.38)	**< 0.0001**
**sCD30, ng/ml**^a^	2.85 (0.54)	2.67 (0.35)	**< 0.0001**	2.85 (0.54)	2.67 (0.35)	**< 0.0001**
**sCD27, U/ml**^a^	3.59 (0.39)	3.46 (0.30)	**< 0.0001**	3.60 (0.40)	3.46 (0.31)	**< 0.0001**
**CXCL13, pg/mL**^a^	3.38 (0.81)	3.08 (0.59)	**< 0.0001**	3.37 (0.81)	3.09 (0.59)	**< 0.0001**
**Lymphoma subtypes, n (%)**						
DLBCL	174 (33.7)	–		174 (33.7)	–	
CLL	210 (40.7)	–		211 (40.8)	–	
FL	132 (25.6)	–		132 (25.5)	–	

^a^Mean (SD); Body mass index (BMI); diffuse large B-cell lymphoma (DLBCL), follicular lymphoma (FL), chronic lymphocytic leukemia (CLL).

^b^one CLL and 9 controls lacked paired samples ; Due to limited sample volume, sCD27 and CXCL13 were not measured in 5 and 11 samples, respectively. So sample size for those markers are as: Matched analyses: CD27 (511 pairs), CXCL13 (506 pairs). Unmatched analyses: CD27 (515 cases/522 controls), CXCL13 (511/520).

^c^Median (min–max). Bold: statistically significant at *p*-value ≤ 0.05.

### Association between immune markers and lymphoma development

Multivariable conditional logistic regression analyses for all BCL cases showed a significant association for all markers when analyzed as categorized variables (Table 2). The subtype-specific analyses rendered similar results except for a non-significantly increased risk of CLL with elevated levels of CXCL13 (Table 2). To account for the correlation between the markers, all markers were also modeled together. The combined multivariable models showed a significant association between sCD23 and all three BCL subtypes, and between CXCL13 and FL and DLBCL (Table 3). Moreover, we found a borderline significant association between sCD27 and all three BCL subtypes.

**Table 2:** Multivariable conditional analyses: odds ratio (OR) and 95% confidence interval (CI) for individual immune marker (categorical variable) and B-cell lymphoma and histological subtypes.

	Marker’s level	BCL		CLL		FL		DLBCL	
		N	OR (95% CI)	N	OR (95% CI)	N	OR (95% CI)	N	OR (95% CI)
**sCD23, pg/mL**									
*Q1*	6.345–7.45	63/130	Ref	16/54	Ref	20/27	Ref	27/49	Ref
*Q2*	7.451–7.72	75/128	1.5 (0.95–2.38)	19/48	1.22 (0.43–2.9)	24/42	0.77 (0.3–1.89)	32/38	1.86 (0.85–4.1)
*Q3*	7.721–7.972	104/128	1.9 (1.25–2.98)	33/51	2.34 (1.03–5.3)	34/33	1.57 (0.7–3.46)	37/44	1.56 (0.7–3.39)
*Q4*	7.973–9.879	274/130	6.2 (3.95–9.74)	142/57	10.5 (4.7–23.4)	54/30	3.88 (1.54–9.8)	78/43	4.10 (1.82–9.2)
*P*		516/516	**< 0.0001**	210/210	**< 0.0001**	132/132	**0.001**	174/174	**0.001**
**sCD30, ng/ml**									
*Q1*	1.555–2.434	98/129	Ref	37/45	Ref	31/40	Ref	30/44	Ref
*Q2*	2.435–2.639	96/128	1.11 (0.74–1.66)	39/52	1.1 (0.49–2.20)	19/34	0.92 (0.4–2.02)	38/42	2.6 (1.15–5.91)
*Q3*	2.640–2.856	122/132	1.36 (0.91–2.06)	52/54	1.42 (0.71–2.8)	27/26	1.56 (0.66–3.6)	43/52	1.99 (0.87–4.6)
*Q4*	2.857–5.621	200/127	2.62 (1.71–3.95)	82/59	2.14 (1.1–4.24)	55/32	3.0 (1.39–6.53)	63/36	5.87 (2.4–14.7)
*P*		516/516	**< 0.0001**	210/210	**0.01**	132/132	**0.003**	174/174	**0.001**
**sCD27, U/ml**									
*Q1*	2.152–3.27	91/128	Ref	35/49	Ref	24/31	Ref	32/48	Ref
*Q2*	3.271–3.459	100/126	1.38 (0.89–2.1)	36/53	1.14 (0.56–2.3)	29/37	1.15 (0.45–2.85)	35/36	1.87 (0.86–4.1)
*Q3*	3.46–3.645	125/131	2.0 (1.25–3.19)	51/51	2.1 (0.98–4.5)	28/33	1.7 (0.61–4.74)	46/47	1.98 (0.85–4.6)
*Q4*	3.646–5.707	195/126	4.24 (2.56–7.0)	85/54	4.63 (2.0–10.7)	50/30	4.29 (1.46–12.7)	60/42	4.32 (1.7–10.9)
*P*		511/511	**< 0.0001**	207/207	**< 0.0001**	131/131	**0.004**	173/173	**0.003**
**CXCL13, pg/mL**									
*Q1*	1.116–2.785	96/129	Ref	52/55	Ref	21/31	Ref	23/43	Ref
*Q2*	2.786–3.086	94/127	1.2 (0.81–1.84)	44/57	0.9 (0.46–1.75)	25/36	1.11 (0.49–2.5)	25/34	1.93 (0.84–4.4)
*Q3*	3.087–3.444	113/125	1.45 (0.95–2.2)	45/39	1.35 (0.7–2.75)	20/32	1.02 (0.4–2.58)	48/54	2.15 (1.0–4.62)
*Q4*	3.445–6.809	203/125	2.6 (1.74–3.94)	63/53	1.39 (0.7–2.7)	62/29	4.01 (1.7–9.37)	78/43	4.67 (2.14–10)
*P*		506/506	**< 0.0001**	204/204	0.17	128/128	**0.0004**	174/174	**< 0.0001**

B-cell lymphoma (BCL), diffuse large B-cell lymphoma (DLBCL), follicular lymphoma (FL), chronic lymphocytic leukemia (CLL); number of cases/controls (N); adjusted for BMI, education, smoking, alcohol intake, and physical activity; Quartiles were calculated based on the distribution in control subjects; tests for trend (*p*) were calculated using the quartile number as a continuous variable. Bold: statistically significant at *p*-value ≤ 0.05.

**Table 3:** Odds ratio (OR) and 95% confidence interval (CI) for combined immune marker (categorical variables) and B-cell lymphoma and histological subtypes.

	OR (95%CI)			p-trend
	Q2	Q3	Q4	
**BCL**				
sCD23	1.46 (0.90–2.37)	1.83 (1.16–2.90)	4.85 (2.98–7.90)	**< 0.0001**
sCD30	1.01 (0.64–1.59)	0.84 (0.53–1.35)	0.99 (0.56–1.66)	0.72
sCD27	1.1 (0.68–1.79)	1.36 (0.80–2.35)	2.35 (1.28–4.30)	**0.001**
CXCL13	1.17 (0.74–1.85)	1.24 (0.78–1.96)	1.96 (1.23–3.14)	**0.001**
**CLL**				
sCD23	1.19 (0.44–3.17)	2.32 (0.99–5.46)	10.3 (4.29–24.8)	**< 0.0001**
sCD30	1.19 (0.48–3.0)	0.80 (0.33–1.96)	0.93 (0.36–2.40)	0.67
sCD27	0.79 (0.32–1.95)	1.09 (0.40–2.95)	1.68 (0.54–2.25)	0.10
CXCL13	0.87 (0.38–2.02)	1.10 (0.45–2.70)	0.80 (0.33–1.91)	0.99
**FL**				
sCD23	0.72 (0.26–1.99)	1.53 (0.62–3.79)	2.81 (0.90–8.75)	**0.02**
sCD30	0.81 (0.32–2.03)	1.39 (0.50–3.88)	1.0 (0.34–2.92)	0.97
sCD27	1.07 (0.34–3.36)	0.97 (0.25–3.73)	2.74 (0.65–11.6)	0.08
CXCL13	0.97 (0.39–2.45)	0.93 (0.33–2.63)	3.41 (1.26–9.22)	**0.01**
**DLBCL**				
sCD23	1.70 (0.71–4.06)	1.19 (0.52–2.74)	2.60 (1.04–6.48)	**0.05**
sCD30	2.33 (0.95–5.75)	1.23 (0.49–3.07)	2.43 (0.83–7.10)	0.41
sCD27	1.40 (0.57–3.41)	1.72 (0.65–4.55)	3.58 (1.15–11.1)	0.08
CXCL13	1.88 (0.78–4.55)	1.84 (0.81–4.19)	3.60 (1.50–8.68)	**0.01**

B-cell lymphoma (BCL), diffuse large B-cell lymphoma (DLBCL), follicular lymphoma (FL), chronic lymphocytic leukemia (CLL); adjusted for BMI, education, smoking, alcohol intake, and physical activity; to see the immune marker levels associated with each quartile see Table 2. Bold: statistically significant at *p*-value ≤ 0.05.

Analyses using continuous measures of the markers showed similar results to the categorical analyses (Supplementary Table 3).

### Analyses stratified by time-to-diagnosis (TTD)

To explore the possibility of reverse causation, associations of the markers with risk of BCL and subtypes were further stratified by median (9 years) duration of time between blood donation and diagnosis of BCL. Unconditional logistic regression among subjects diagnosed ≤ 9 years from the time of blood collection essentially showed similar associations as presented in Table 2. Subjects diagnosed more than 9 years from the time of blood collection with elevated levels of sCD23 and sCD27 showed an increased risk of CLL, while increased levels of all markers were associated with higher risk of DLBCL (Supplementary Table 4). In the combined model (including all markers together), associations of sCD23 with CLL and CXCL13, sCD23, and sCD27 with DLBCL remained significant.

**Figure 1:**
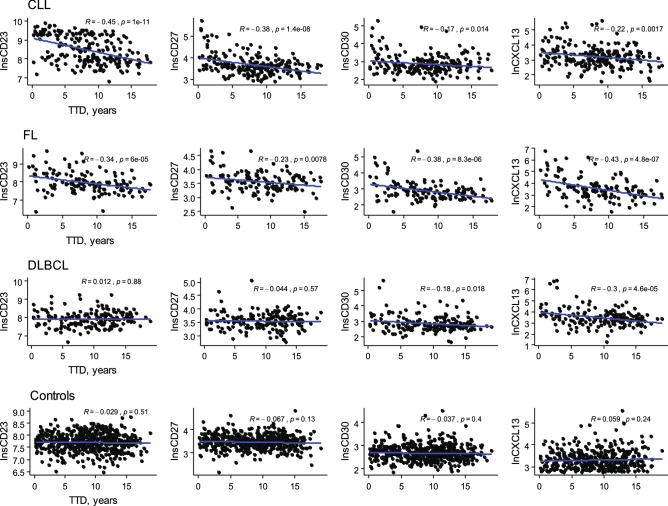
Correlation between serum level of immune markers and time to diagnosis (TTD) for different BCL subtype and controls; TTD for controls was calculated based on diagnosis date of the index cases.

The correlation between marker levels and time to diagnosis was evaluated. These analyses revealed higher levels of the markers in those cases with blood drawing closer to the diagnosis date (Fig. 1) except for sCD23 and sCD27 among DLBCL cases, which may suggest that serum levels of these markers are impacted by the disease itself.

### Receiver operating characteristic (ROC) and test performance analysis

The AUC, as a measure of how well a marker (or a group of markers) predict the development of BCL subtype, was calculated by tenfold cross-validation. We found an AUC of 0.80 for a model including sCD23 as a predictor of CLL. Addition of other markers to the model did not significantly increase the prediction ability for CLL (Supplementary Table 5). sCD23 levels showed the highest prediction ability for CLL among male participants (AUC = 0.85, *p* = 0.0003) and more particularly those older than 60 years as indicated by an AUC 0.88 (*p* = 0.02) (Supplementary Table 6). sCD23 and CXCL13 showed a lower predictive ability for FL (AUC ~ 0.60) and DLBCL (AUC ~ 0.63) compared with CLL and addition of other markers did not significantly increase the AUCs for these subtypes (Supplementary Table 5).

**Figure 2:**
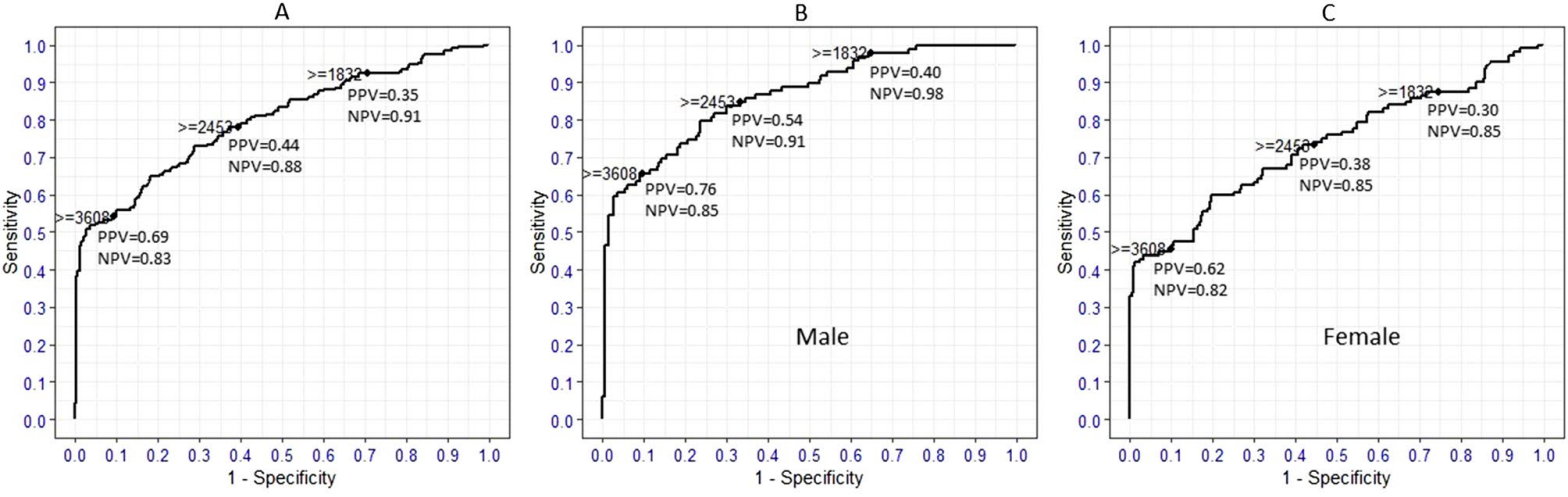
ROC curve and test accuracy parameters of sCD23 for cutoff at 30th (≥ 1832 pg/mL), 60th (≥ 2,453 pg/mL), and 90th (≥ 3,608 pg/mL) deciles for CLL (**A**) and stratified by gender (**B**, **C**); *ROC* receiver operating characteristic, *PPV* positive predictive value, *NPV* negative predictive value.

While the AUCs gave an overall picture of the behavior of the markers across all cutoff values, there remains a practical need to determine the specific cutoff value that could be used for individuals requiring screening. Therefore, sensitivity, specificity, positive predictive value (PPV), and negative predictive value (NPV) for different cut-off values of sCD23 for the prediction of CLL, FL, and DLBCL and cut-off values of CXCL13 for the prediction of FL and DLBCL were determined (Supplementary Table 7–8). Generally, there is a trade-off between sensitivity and specificity. Using the cut-off value showing the highest specificity, subjects with sCD23 levels ≥ 3,608 pg/mL appeared to have a 70% probability to develop CLL after 9 years (Fig. 2a, Supplementary Table 7), the probability being slightly higher for male (0.76) than for female (0.62) participants (Fig. 2b, c, Supplementary Table 7). When analyzing sCD23 and CXCL13 test accuracy for DLBCL and FL, it was observed that despite the specificity of sCD23 being the same for FL and DLBCL, PPVs were low compared to CLL (Supplementary Table 8).

### Mediation analyses

Finally, to evaluate the hypothesis that immune markers act as a mediator on the causal pathway between known risk factors and B-cell lymphoma, a causal mediation analysis was conducted. Actual analyses were performed if (1) the risk factors were significantly associated with immune markers (Supplementary Table 9, model M) and (2) immune markers were found to be significantly associated with lymphoma subtypes in our combined models (Table 3). Selection of possible risk factors for mediation analyses was based on a large pooled study from 20 case–control studies^13^. Although, most of these associations were only suggestive and not significant in EPIC (Model X in Supplementary Table 9), the cohort can still help to understand potential mechanisms due to the prospective framework. Therefore, results of the mediation analysis should be seen as suggestive mediated associations. Positive association between BMI and DLBCL (average causal mediation effect (ACME) = 0.02) mediated through both sCD23 and CXCL13 (Table 4). We found a lower risk of DLBCL (ACME = − 0.02) with physical activity mediated through CXCL13. Finally, there was a trend toward significance for a protective effect of alcohol intake (ACME = − 0.05) mediated through sCD23 on CLL and for a protective effect of physical activity (ACME = − 0.01) mediated through CXCL13 on FL (Table 4). Sensitivity analysis were conducted to evaluate the robustness of the results from the causal mediation analysis. The analyses showed that as long as ρ was 0.4 or lower, the estimated mediated effects still had the same sign, indicating good robustness.

**Table 4:** Average direct effect and causal mediation effect (mediated through B-cell activation markers) of known risk factors on B-cell lymphoma subtypes.

	Average causal mediation effect	ρ	Average direct effect *	Total effect	Proportion mediated ‡
**sCD23, CLL, n = 737**					
BMI	0.04 (− 0.006 to 0.09)	0.4	0.05 (− 0.03 to 0.12)	0.09 (− 0.01 to 0.18)†	0.44
Alcohol	− 0.05 (− 0.10 to 0.003)†	0.4	0.01 (− 0.07 to 0.09)	− 0.04 (− 0.14 to 0.06)	0.80
**sCD23, DLBCL, n = 698**					
BMI	**0.02 (0.002 to 0.04)**	0.3	− 0.07 (− 0.14 to 0.02)	− 0.05 (− 0.12 to 0.04)	− 0.26
**CXCL13, FL, n = 648**					
Physical activity	− 0.01 (− 0.03 to 0.001)†	0.3	0.002 (− 0.07 to 0.08)	− 0.01 (− 0.08 to 0.06)	0.23
**CXCL13, DLBCL, n = 693**					
BMI	**0.02 (0.0005 to 0.05)**	0.4	− 0.07 (− 0.15 to 0.02)	− 0.05 (− 0.12 to 0.04)	− 0.34
Physical activity	− **0.02 (**− **0.05 to **− **0.003)**	0.3	0.01 (− 0.07 to 0.09)	− 0.01 (− 0.09 to 0.07)	0.41

Diffuse large B-cell lymphoma (DLBCL), follicular lymphoma (FL), chronic lymphocytic leukemia (CLL); Cases and control have been analyzed together; Correlation parameter (ρ) for mediated effect at which the sign of average causal mediation effect (ACME) is reversed; Effect estimates are the change in probability for moving the exposure variable from the reference category to the exposure category: alcohol intake: non-drinker (0) versus drinker (1), activity: first 3 categories (0) versus active(1), and BMI: < 30 (0) versus ≥ 30 (1); * “average direct effect” shows the estimate for unmediated effect which is the effect from any other path except the mediated one; † significant at p = 0.065; ‡ Shows the percentage of total effect mediated by the immune marker, it can be negative or greater than 100 depend on direct and mediated effect magnitudes and directions. Bold: statistically significant at *p*-value ≤ 0.05.

## Discussion

In this prospective study, we determined the serum levels of previously reported lymphoma associated immune markers and investigated how these markers correlate to the future risk of BCL histological subtypes and if risk of lymphoma is mediated by these markers. From these results, we confirmed the association between pre-diagnostically measured B-cell activation markers (sCD23, sCD27, sCD30, and CXCL13) and subsequent development of major BCL subtypes^8–12,28,29^. After adjustment for other immune markers, sCD23 remained significantly associated with all subtypes, while CXCL13 was found to be associated with FL and DLBCL only. The associations between sCD23 and CLL and DLBCL, and CXCL13 and DLBCL persisted among cases sampled more than 9 years before diagnosis, although the associations were attenuated in comparison to the findings from the models that covered the entire follow-up. sCD23 showed the highest prognostic value for CLL. In addition, we assessed for the first time, whether these markers may be mediating the causal pathway between several risk/protective factors and later lymphoma risk. Our results suggest that sCD23 and CXCL13 partly mediated the association between BMI (positive) and DLBCL risk, while lower levels of CXCL13 associated with higher physical activity and this partly explains the inverse association of physical activity and DLBCL risk. It should be noted that the measured biomarkers are individual markers in a complicated signaling milieu. Therefore any reported mediating effect for the marker itself should be interpreted as a proxy of the underlying biologic milieu that is captured by circulating levels of those molecules that is partly mediating the alleged associations.

We observed strong associations between sCD23, sCD27, sCD30, and CXCL13 in blood samples collected up to 19 years before diagnosis and risk of BCL development, consistent with previous studies^8–12,28,29^. The associations between sCD23 levels and development of BCL subtype were the most stable associations in this study, particularly for CLL, which was significant even more than 9 years before BCL diagnosis. In a recent study using the EPIC cohort with maximum 12.5 years TTD, ROC curve for the prediction or diagnosis of CLL indicates that sCD23 is a strong predictive marker (AUC = 0.88)^30^ which is consistent with our current finding of AUC = 0.80. The earlier study included 179 CLL cases that were also present in our study. AUC based on new cases only (maximum 19 years follow-up) was 0.73. Moreover, our study showed that sCD23 is apparently more predictive in men than in women for future risk of CLL. Little is known how sex-specific factors influence CLL incidence. Although androgen receptors play a part in lymphopoiesis, it is unclear how this relates to the sex differences in CLL. As second explanation, sex-specific somatic alterations in the non-pseudoautosomal and pseudoautosomal regions on chromosomes X and Y have also been suggested to influence CLL incidence^30^.

CD23 is an integral membrane glycoprotein involved in IgE binding, and is found on mature B-cells, activated macrophages, eosinophils, follicular dendritic cells, and platelets. CD23 is expressed on the membrane in CLL, in some cases of FL, and primary mediastinal large B-cell lymphoma. CLL cells have a characteristic phenotype of sIg^low^/CD19^+^/CD5^+^/CD23^+^^31^. CD23 has been widely used as a marker in the differential diagnosis of CLL versus mantle cell lymphoma. Its soluble form, sCD23, is released from activated B-cells and can itself induce further B-cell stimulation as well as function as a potent mitogenic growth factor. Many reports suggest that elevated CD23, either on neoplastic cell surfaces or as a soluble form, is a useful marker in either diagnosis or prognosis of BCL^31^. Assuming that increased levels of sCD23 are caused by early stages of disease, our results further support that sCD23 would act as a marker for early detection of CLL^28^.

In a study within the Northern Sweden Health and Disease Study (NSHDS), B-cell activation markers were measured in two pre-diagnostic blood samples donated by 170 individuals before BCL diagnosis, along with 170 matched cancer-free controls^29^. The study showed that regardless of baseline B-cell activation marker concentration, BCL future risk was also associated with an increase of marker concentrations over time (slope). The predictive ability of these markers for response to treatment as well as their prognostic value for disease progression in particular in the trajectory from monoclonal B-cell lymphocytosis (MBL), an asymptomatic condition in which small numbers of clonal B cells are detectable in blood, to CLL must be further assessed.

CXCL13 is a CXC subtype member of the chemokine superfamily and acts via its receptor CXCR5 as one of the most potent B-cell chemo-attractants. CXCL13 expression is observed in BCL and diseases with B-cell activation. We reported that higher levels of CXCL13 were associated with increased risk of DLBCL, independently of sCD23, even among subjects diagnosed > 9 years after study initiation. Our finding is consistent with a previous report within the NSHDS (42 DLBCL cases)^29^, the Women’s Health Initiative Observational Study cohort (138 DLBCL cases)^8^, and Nurses’ Health Study and Health Professionals Follow-up Study (107 DLBCL cases)^32^. This would argue against the idea that increased marker concentrations would be attributable to undiagnosed disease, considering that the median survival of DLBCL is expected to be only a few months if left untreated^29^.

The fact that measured B-cell activation marker levels were higher in those cases with blood drawing closer to the diagnosis date may indicate the existence of undiagnosed lymphoproliferative disease, in particular for indolent BCL subtypes. On the other hand, this may also reflect biological processes involved in the onset of the disease, e.g. as a measure of the allostatic load on the B-cell compartment related to non-heritable factors such as lifestyle and environmental factors^18–25^. Previous studies showed that elevated concentrations of these markers were associated with disease states related to immune system activation, such as autoimmune diseases, hepatitis C, and HIV infection^18–23^. However, such information was not available on our subjects, except for data on HBV infection from only a minority of the subjects (n = 335).

Currently, no established screening programs for BCL development exist. Pre-diagnostic screening for risk factors of BCL in the general population has currently little clinical benefit. Moreover, cut-offs of marker levels to be used as a measure for disease progression have not been established yet. This would require the conduct of a large number of studies within various populations in order to establish normal levels and resulting in a more uniform reference for “abnormal” levels for use in clinical practice. In our view, the value of pre-diagnostic biomarkers is potentially much larger in groups of patients that are already at elevated risk of developing BCL, such as those with a family history of lymphoma and immunocompromised patients (i.e., primary immunodeficiency disorders, PID). PID patients carry an eightfold increased risk of lymphoma compared to the general population. Patients who have higher levels of the markers may thus be recommended to start intensive follow-up, while individuals with lower level of the markers may be advised for a less intensive follow-up. However, usefulness of these markers must be examined and validated among those patients before they could be rationally employed by physicians to improve human health.

To our knowledge, this is the first study to assess the hypothesis of B-cell activation markers as mediators on the potential pathway between risk factors and later lymphoma development. BMI was associated with a significant increased risk of DLBCL mediated through sCD23 and CXCL13. There is no clear consensus on how obesity impacts B-cell development and function, but several studies point to an involvement of B-cells in adipose tissue inflammation associated with obesity^33–35^. A further study in obese and non‐obese women revealed that body fat mass was positively correlated with total leukocyte, neutrophil, monocyte and lymphocyte counts^36^. While T‐cell function was comparable between obese and non‐obese women, B-cell function was about 50% higher in the obese group.

Physical activity was associated with decreased risk of DLBCL and FL mediated through CXCL13. There has been evidence that moderate exercise is beneficial for the immune system^37^. Exercise induced changes in the number and function of cell subsets involved in the innate (e.g., neutrophils, monocytes, and natural killer cells) and the adaptive immune system (e.g., T and B cells)^37,38^. It has been shown that exercise can reduce insulin, glucose, and insulin-like growth factors, which may influence the proliferation of tumor cells in general. Physical activity also plays role in the prevention of obesity and reduces the percentage of body fat^38^. However, it is not clear if modulation of the immune system contributes to the potential antitumor properties of exercise.

Adaptive immune responses, also called acquired immunity, is characterized by antigen‐specific T‐cell proliferation, immunological memory, B-cell activation, and production of antibodies. Evidence indicates that alcohol exposure can interfere with various aspects of the immune response and affect the different cellular components of the innate and/or adaptive immune system. Several studies reported that the number and function of B-cells are reduced by alcohol^26,27^. In our study, sCD23 mediated the association between alcohol intake and decreased risk of CLL. A meta-analysis provided evidence for a favorable role of alcohol drinking on NHL risk^39^. However, there is no clear biological explanation for this association.

A strength of our study is the relatively large number of incident cases of newly diagnosed major BCL subtypes in a cohort of cancer-free individuals with pre-diagnostic blood samples and prospective follow-up times of up to 19 years. This enabled us to carry out specific analyses according to BCL subtypes. This is particularly relevant since there is growing evidence that lymphoma subtypes have different pathological and epidemiological features^13^. However, limitations of our study should be considered when interpreting the results. We measured blood immune markers at a single time point, which may not reflect accurately the long-term B-cell activation status and may not capture the most important etiologic timing. We cannot exclude potential measurement errors derived from dietary questionnaires, which could lead to systematic and random errors when estimating alcohol intake. Likewise, anthropometric measures were ascertained at recruitment (with the exception of the Oxford, France and Norway cohorts—self-reported). Moreover, we cannot exclude the possible bias due to unmeasured confounders (e.g., immune diseases and infections), which are well known risk factors of BCL. If an unmeasured confounder is related to several mediators this may have resulted in a bias in the mediation results^40^. Due to the limited sample size, the results of this sensitivity analysis should be interpreted with caution. Notably, the measured immune markers are not only produced by those cell types considered to play pivotal roles in the immune system (lymphocytes), but also by fibroblasts, neutrophils, eosinophils, follicular dendritic cells, and platelets. So, blood levels of the markers may not necessarily reflect activity in the target tissue (lymph nodes)^41^.

In conclusion, increased B-cell activation marker levels present in blood years before BCL diagnosis, suggest a role of B-cell activation in BCL development at early stages. These may reflect a constitutional predisposition with shared underlying mechanisms for both indolent and aggressive lymphoma subtypes. Further studies investigating the biological and clinical impact of these markers are required. The mediating role of the immune function in the association between lifestyle factors and BCL also needs further examination.

## Materials and methods

### Study population

The EPIC study is a prospective cohort study involving 23 centers from ten European countries (Denmark, France, Germany, Greece, the Netherlands, Italy, Norway, United Kingdom, Spain and Sweden) that was designed to investigate the potential relationships between diet, nutritional status, lifestyle and environmental factors and the incidence of cancer and other chronic diseases^42^. Over 500,000 healthy subjects in the age range 35–70 were recruited in the study during 1992–2000. The rationale, complete methodology and study design of the EPIC study have been described previously^42,43^. The ethical review boards from the International Agency for Research on Cancer and all local participating centers approved the study and all participants gave their informed consent. The study was conducted in accordance with the approved guidelines.

Standardized lifestyle, medical and personal history, and diet questionnaires were collected from the participants, and a blood sample was taken at enrollment. Anthropometric measures were measured at recruitment (except for France, Oxford and Norway who collected self-reporting data). Within 2 h of blood collection, blood samples were processed for the isolation of buffy coats and other fractions. Samples were transported on dry ice to the laboratory and stored at − 80 °C before analyses were performed.

Incident lymphoma cancer cases were identified by population cancer registries for Denmark, Italy, The Netherlands, Norway, Spain, Sweden and the United Kingdom. A combination of methods was used in France, Germany and Greece, as detailed previously^42^. Originally, the diagnosis of lymphoma cases was based on the ICD-O-2. All lymphoma cases were subsequently reclassified according to the SEER ICD-O-3 morphology codes^44^. For each incident BCL case identified within the cohort by December 2012, one random control was selected among all cohort members alive and free of cancer at the time of diagnosis of the index case, matched by country, center, gender, age at recruitment (± 12 months), date of blood collection (± 3 months), time of blood collection (± 3 h), and fasting at blood collection.

The current analysis was based on 516 case–control pairs for which a blood sample was available consisting of 174 DLBCL, 132 FL, and 210 CLL (including small lymphocytic leukemia) cases. For one CLL case and 9 controls, paired samples were missing. These subjects were included only in unconditional logistic analyses.

### Inclusion of etiological factors for FL, DLBCL, CLL subtypes

Previously known BCL risk and protective factors^13^ that were available in EPIC were included in the study. These included BMI, height, smoking, education as proxy for SEP, alcohol intake and physical activity. The physical activity assessment included occupation as well as average recreational and household activity.

### Measurement of immune markers

Serum levels of sCD23, sCD27, sCD30, and CXCL13 were measured by ELISA for all samples (eBioscience, USA: BMS286INST, BMS240INST kits, and R&D Systems, USA: DCD230 and DCX130 kits). Assays were performed in duplicate and according to the manufacturers’ protocols. All laboratory personnel were blinded with regard to case–control status. Matched case–control sets were assayed next to each other on the same plate in the same batch and quality control samples were run in duplicate along with the case–control sets in each batch. Inter- and intra-assay coefficients of variation were 2.2% and 3.1% for sCD23, 8.9% and 8.8% for sCD27, 4.8% and 7.3% for sCD30, and 5.2% and 6.8% for CXCL13.

### Statistical analysis

Marker levels measured out of range of the calibration curve (sCD23 = 6.3%, sCD30 = 0.8%, sCD27 = 0.5%, CXCL13 = 19.1%) and missing values of smoking status (2%), education (7%), alcohol intake (0.4%), physical activity (2.7%) covariates (Supplementary Table 10) were imputed based on a maximum likelihood estimation method which was informed by the observed correlation structure within the data^45^. Blood levels of soluble markers were log transformed to normalize their distributions. Differences between cases and controls in baseline continuous covariates were assessed using the paired *t *test, and by *χ*^2^-test, for categorical variables. Spearman rank correlation was used to measure the degree of correlation between markers.

Odds ratios (OR) and 95% confidence intervals (95% CI) for the subtypes of BCL in relation to immune markers (as continuous variables) were calculated by conditional logistic regression (CLR). The models were adjusted for BMI (kg/m^2^, continuous), alcohol intake (g/day; continuous), smoking status (never, former, current), physical activity levels based on the Cambridge Physical Activity Index (inactive, moderately inactive, moderately active, active)^46^ and educational level (none, primary, technical/professional, secondary, university/college). Quartiles of immune marker concentrations were calculated based on the distribution in control subjects, and CLR models were used to estimate the association between quartiles of marker levels and risk of BCL subtypes (first quartile as reference category). Tests for trend were calculated using the quartile number as a continuous variable. All markers were also modeled together (combined multivariable model) as it may be possible that one marker serves as a surrogate of another.

Associations of the markers with risk of BCL and subtypes were additionally stratified by median duration of time between blood donation and diagnosis of BCL (time-to-diagnosis: TTD) to explore the possibility of reverse causation. In these analyses, to preserve statistical power, subtype cases were compared to all controls and (unconditional) models additionally adjusted for matching variables (i.e., country, gender, and age at recruitment) and plate number.

Receiver operating characteristic (ROC) analysis and AUC comparisons were used to determine the discriminative ability of the markers separately or in combination with other markers. The AUCs was corrected for biases due to overfitting by tenfold cross-validation. For each fold, the AUC was calculated, and the mean of the fold AUCs was the cross-validated AUC estimate. Four objective measures of test performance were further calculated, namely, sensitivity, specificity, positive predictive value (PPV) and negative predictive value (NPV) for different cut-off values of the markers found to be informative for future risk of BCL^47^. Cut-off values were calculated based on deciles of ranked level of the markers in control subjects.

Causal mediation analysis was applied to study the average causal mediation effect (ACME) and the average direct (unmediated) effect (ADE) of immune markers linking risk factors to lymphoma^48^. The effect estimates represent the change in probability that the subject develops lymphoma when moving the exposure variable from the reference category to the exposure category via the mediated or direct paths. Further, the analysis provides an estimate of the proportion of the total effect of exposure on lymphoma development mediated through the measured marker. Included exposure variables were smoking: non-smoker at recruitment (0) vs. smoker (1), alcohol intake: non-drinker (0) vs. drinker (1), physical activity: first 3 categories (0) vs. active(1), education: categories primary school or lower/ technical/ vocational school (0) vs. secondary school/ university/college (1), BMI: < 30 (0) versus ≥ 30 (1), and height: < country median (0) versus ≥ country median (1). We fitted two statistical models, the mediator (*M*) linear model for the conditional distribution of the mediator *M* given the risk factor *X* and a set of the covariates *C*; *f(M | X, C),* and the outcome (*Y*) logistic model for the conditional distribution of the outcome *Y* given *X*, *M* , and *C; f(Y* | *X, M, C).* These models were fitted separately and then their fitted objects comprised the main inputs to the *mediate* function, which computes the estimated ACME and other quantities of interest under these models and the sequential ignorability assumption. Mediation analyses were applied only for the risk factors significantly associated with immune markers and for the immune markers found to be significantly associated with lymphoma subtypes in our combined models (sCD23 and CXCL13). Adjustments were made for country, sex, and age. Models for each risk factor were additionally adjusted for other risk factors. There was no significant interaction between the risk factors and sCD23 and CXCL13. Quasi-Bayesian confidence intervals were determined^49^. Sensitivity analyses were performed for deviations from the sequential ignorability assumption (that in particular implies no unmeasured pre-sample collection confounders), with deviations measured by the correlation ρ between the errors in the mediation and the outcome models. In the presence of confounders which affect both the mediator and the outcome, we expect that the sequential ignorability assumption is violated and ρ is no longer zero^48^. A large critical ρ value reversing the sign of ACME indicates the violation of ignorability assumption^49^.

Statistical analyses were performed using the R 3.4.1 language and environment (The R Foundation for Statistical Computing, Vienna, Austria) and SAS (version 9.4; SAS institute, USA). The R package mediation (4.1.2) was used for causal mediation analysis^49^. All *p* values are two-sided, with *p* < 0.05 considered as statistically significant.

## Supplementary information

Supplementary Information.

## Data Availability

The research protocol, data analysis plan, syntaxes, and analysis files can be available from the corresponding author. Data may not be shared as the EPIC explicitly retains ownership of the primary data and we agreed not to transfer the primary data disclosed under our agreement with the data owner to any third parties.

## References

[1] Ambinder RF, Bhatia K, Martinez-Maza O, Mitsuyasu R (2010). Cancer biomarkers in HIV patients. Curr. Opin. HIV AIDS.

[2] Breen EC (2011). B-cell stimulatory cytokines and markers of immune activation are elevated several years prior to the diagnosis of systemic AIDS–associated non-hodgkin B-Cell lymphoma. Cancer Epidemiol. Biomark. Prev..

[3] Breen EC (2006). Elevated serum soluble CD30 precedes the development of AIDS-associated non-Hodgkin’s B cell lymphoma. Tumour Biol..

[4] Widney D (1999). Aberrant expression of CD27 and soluble CD27 (sCD27) in HIV infection and in AIDS-associated lymphoma. Clin. Immunol..

[5] Widney DP (2005). Serum levels of the homeostatic B cell chemokine, CXCL13, are elevated during HIV infection. J. Interferon Cytokine Res..

[6] Widney DP (2010). Expression and function of the chemokine, CXCL13, and its receptor, CXCR5, in AIDS-associated non-Hodgkin's lymphoma. AIDS Res. Treat..

[7] Breen EC (2005). Elevated levels of soluble CD44 precede the development of AIDS-associated non-Hodgkin's B-cell lymphoma. AIDS.

[8] De Roos AJ (2012). Markers of B-cell activation in relation to risk of non-Hodgkin lymphoma. Cancer Res..

[9] Purdue MP (2011). Prediagnostic serum levels of cytokines and other immune markers and risk of non-Hodgkin lymphoma. Cancer Res..

[10] Purdue MP (2009). A prospective study of serum soluble CD30 concentration and risk of non-Hodgkin lymphoma. Blood.

[11] Vermeulen R (2011). Circulating soluble CD30 and future risk of lymphoma; evidence from two prospective studies in the general population. Cancer Epidemiol. Biomark. Prev..

[12] Hosnijeh FS (2016). Soluble B-cell activation markers sCD27 and sCD30 and future risk of B-cell Lymphomas and multiple myeloma: a nested case-control study and meta-analyses. Int. J. Cancer.

[13] Morton LM (2014). Etiologic heterogeneity among non-Hodgkin lymphoma subtypes: the InterLymph non-Hodgkin lymphoma subtypes project. J. Natl. Cancer Inst. Monogr..

[14] Psaltopoulou T (2019). Anthropometric characteristics, physical activity and risk of hematological malignancies: a systematic review and meta-analysis of cohort studies. Int. J. Cancer.

[15] Hosnijeh FS, Heederik D, Vermeulen R (2012). A review of the role of lymphoma markers and occupational and environmental exposures. Vet. Q..

[16] Skibola CF (2007). Obesity, diet and risk of non-Hodgkin lymphoma. Cancer Epidemiol. Biomark. Prev..

[17] Lam QL, Wang S, Ko OK, Kincade PW, Lu L (2010). Leptin signaling maintains B-cell homeostasis via induction of Bcl-2 and Cyclin D1. Proc. Natl. Acad. Sci. U. S. A..

[18] Schneider C, Hübinger G (2002). Pleiotropic signal transduction mediated by human CD30: a member of the tumor necrosis factor receptor family. Leuk. Lymphoma.

[19] Monsalve F, Romero-A T, Estevez J, Costa L, Callejas D (2001). Serum levels of soluble CD30 molecule in hepatitis B virus infection. Rev. Med Chile.

[20] Fattovich G (1996). Serum levels of soluble CD30 in chronic hepatitis B virus infection. Clin. Exp. Immunol..

[21] Bansal AS, Bruce J, Hogan PG, Prichard P, Powell EE (1997). Serum soluble CD23 but not IL8, IL10, GM-CSF, or IFN-gamma is elevated in patients with hepatitis C infection. Clin. Immunol. Immunopathol..

[22] Al-Janadi M, Al-Wabel A, Raziuddin S (1994). Soluble CD23 and interleukin-4 levels in autoimmune chronic active hepatitis and systemic lupus erythematosus. Clin. Immunol. Immunopathol..

[23] Terrier B (2014). Serum biomarker signature identifies patients with B-cell non-Hodgkin lymphoma associated with cryoglobulinemia vasculitis in chronic HCV infection. Autoimmun. Rev..

[24] Lan Q (2010). Occupational exposure to trichloroethylene is associated with a decline in lymphocyte subsets and soluble CD27 and CD30 markers. Carcinogenesis.

[25] Hosnijeh FS, Portengen L, Bueno-de-Mesquita HB, Heederik D, Vermeulen R (2013). Circulating soluble CD27 and CD30 in workers exposed to 2,3,7,8-tetrachlorodibenzo-p-dioxin. Cancer Epidemiol. Biomark. Prev..

[26] Chen D, Zhang F, Ren H, Luo J, Wang S (2017). Role of cytokines and chemokines in alcohol-induced tumor promotion. Onco Targets Ther..

[27] Pasala S, Barr T, Messaoudi I (2015). Impact of alcohol abuse on the adaptive immune system. Alcohol Res..

[28] Kaaks R (2015). Lag times between lymphoproliferative disorder and clinical diagnosis of chronic lymphocytic leukemia: a prospective analysis using plasma soluble CD23. Cancer Epidemiol. Biomark. Prev..

[29] Späth F (2017). Biomarker dynamics in B-cell lymphoma: a longitudinal prospective study of plasma samples up to 25 years before diagnosis. Cancer Res..

[30] Allain EP (2018). Sex-dependent association of circulating sex steroids and pituitary hormones with treatment-free survival in chronic lymphocytic leukemia patients. Ann. Hematol..

[31] Acharya M (2010). CD23/FcεRII: molecular multi-tasking. Clin. Exp. Immunol..

[32] Epstein MM (2018). Pre-diagnosis plasma immune markers and risk of non-Hodgkin lymphoma in two prospective cohort studies. Haematologica.

[33] Shaikh SR, Haas KM, Beck MA, Teague H (2014). The effects of diet-induced obesity on B cell function. Clin. Exp. Immunol..

[34] DeFuria J (2013). B cells promote inflammation in obesity and type 2 diabetes through regulation of T-cell function and an inflammatory cytokine profile. Proc. Natl. Acad. Sci. U. S. A..

[35] Marti A, Marcos A, Martinez JA (2001). Obesity and immune function relationships. Obes. Rev..

[36] Nieman DC (1996). Immune response to obesity and moderate weight loss. Int. J. Obes. Relat. Metab. Disord..

[37] Walsh NP (2011). Position statement. Part one: immune function and exercise. Exerc. Immunol. Rev..

[38] Vermaete NV (2013). Physical activity and risk of lymphoma: a meta-analysis. Cancer Epidemiol. Biomark. Prev..

[39] Psaltopoulou T (2018). Alcohol consumption and risk of hematological malignancies: a meta-analysis of prospective studies. Int. J. Cancer.

[40] Jerolon, A., Baglietto, L., Birmele, E., Perduca, V. & Alarcon, F. Causal mediation analysis in presence of multiple mediators uncausally related. arXiv:1809.08018v2 [stat.ME] (2018). 10.1515/ijb-2019-0088 32990647

[41] Hosnijeh FS (2010). Plasma cytokines and future risk of non-Hodgkin lymphoma (NHL): a case-control study nested in the Italian European prospective investigation into cancer and nutrition. Cancer Epidemiol. Biomark. Prev..

[42] Riboli E (2002). European prospective investigation into cancer and nutrition (EPIC): study populations and data collection. Public Health Nutr..

[43] Riboli E, Kaaks R (1997). The EPIC Project: rationale and study design. European prospective investigation into cancer and nutrition. Int. J. Epidemiol..

[44] Fritz A, Percy C, Jack A, Shanmugaratnam K, Sobin L, Parkin DM, Whelan S (2000). International classification of diseases for oncology (ICD-O).

[45] Lubin JH (2004). Epidemiologic evaluation of measurement data in the presence of detection limits. Environ. Health Prospect..

[46] Wareham NJ (2003). Validity and repeatability of a simple index derived from the short physical activity questionnaire used in the European Prospective Investigation into Cancer and Nutrition (EPIC) study. Public Health Nutr..

[47] Maceneaney PM, Malone DE (2000). The meaning of diagnostic test results: a spreadsheet for swift data analysis. Clin. Radiol..

[48] Imai K, Keele L, Tingley D (2010). A general approach to causal mediation analysis. Psychol. Methods.

[49] Tingley D, Yamamoto T, Hirose K, Keele L, Imai K (2014). Mediation: R Package for causal mediation analysis. J. Stat. Softw..

