# Supplementary material for: Mediating effect of soluble B-cell activation immune markers on the association between anthropometric and lifestyle factors and lymphoma development
Source: Sci Rep. 2020 Aug 14;10:13814. doi: 10.1038/s41598-020-70790-9 (PMC7429856; doi:10.1038/s41598-020-70790-9)
Supplement: Supplementary file 1 — Supplementary Information. [file 41598_2020_70790_MOESM1_ESM.docx]

**Mediating effect of soluble B-cell activation immune markers on the association between anthropometric and lifestyle factors and lymphoma development**

Fatemeh Saberi Hosnijeh^1,2^, Pieter M. Kolijn^1^, Delphine Casabonne^3,4^, Alexandra Nieters^5^, Marta Solans ^3,6^, Sabine Naudin^7^, Pietro Ferrari^7^, James D. Mckay^8^, Elisabete Weiderpass^9^, Vittorio Perduca^10,11^, Caroline Besson^11-13^, Francesca Romana Mancini^11,12^, Giovanna Masala^14^, Vittorio Krogh^15^, Fulvio Ricceri^16,17^, José M. Huerta^18,19^, Dafina Petrova^19-21^, Núria Sala^22^, Antonia Trichopoulou^23^, Anna Karakatsani^23,24^, Carlo La Vecchia^23,25^, Rudolf Kaaks^26^, Federico Canzian^27^, Dagfinn Aune^28-30^, Heiner Boeing^31^, Matthias B. Schulze^32,33^, Aurora Perez-Cornago^34^, Anton W. Langerak^1^, Vincent H.J. van der Velden^1^, and Roel Vermeulen^2,35,36^

**Affiliations:**

^1^ Department of Immunology, Laboratory Medical Immunology, Erasmus MC, University Medical Center, Rotterdam, the Netherlands

^2^ Institute for Risk Assessment Sciences, Division of Environmental Epidemiology, Utrecht University, Utrecht, the Netherlands

^3^ Centro de Investigación Biomédica en Red de Epidemiología y Salud Pública, M.P. (CIBERESP), Madrid, Spain

^4^ Unit of Infections and Cancer, Cancer Epidemiology Research PRogramme, Catalan Institute of Oncology, IDIBELL, l'Hospitalet de Llobregat, Spain

^5^ Institute for Immunodeficiency, Faculty of Medicine and Medical Center, University of Freiburg, Freiburg, Germany

^6^ Research Group on Statistics, Econometrics and Health (GRECS), University of Girona, Girona, Spain.

^7^ Nutritional Methodology and Biostatistics Group, International Agency for Research on Cancer, World Health Organization, Lyon, France

^8^ Section of Genetics, International Agency for Research on Cancer, Lyon, France

^9^ International Agency for Research on Cancer– World Health Organization, Lyon, France

^10^ Université de Paris, CNRS, MAP5 UMR 8145, F-75006 Paris, France

^11^ CESP, Fac. de Médecine - Univ. Paris-Sud, Fac de Médecine - UVSQ, INSERM, Université Paris Saclay, 94805, Villejuif FR

^12^ Gustave Roussy, F-94805, Villejuif, France

^13^ Department of Hematology and Oncology, Hospital of Versailles, Le Chesnay, France

^14^ Cancer Risk Factors and Life-Style Epidemiology Unit, Institute for Cancer Research, Prevention and Clinical Network - ISPRO, Florence, Italy

^15^ Epidemiology and Prevention Unit, Fondazione IRCCS Istituto Nazionale dei Tumori di Milano, Milan, Italy

^16^ Department of Clinical and Biological Sciences, University of Turin, Turin, Italy

^17^ Unit of Epidemiology, Regional Health Service ASL, Turin, Italy

^18^ Department of Epidemiology, Murcia Regional Health Council, IMIB-Arrixaca, Murcia, Spain.

^19^ CIBER of Epidemiology and Public Health (CIBERESP), Madrid, Spain

^20^ Andalusian School of Public Health (EASP), Granada, Spain

^21^ Instituto de Investigación Biosanitaria de Granada (ibs.GRANADA), Universidad de Granada. Granada, Spain

^22^ Unit of Nutrition, Environment and Cancer, Cancer Epidemiology Research Program and Translational Research Laboratory, Catalan Institute of Oncology (ICO), Biomedical Research Institute (IDIBELL), Barcelona, Spain

^23^ Hellenic Health Foundation, Athens, Greece

^24^ Pulmonary Medicine Dept., School of Medicine, National and Kapodistrian University of Athens, “ATTIKON” University Hospital, Haidari, Greece

^25^ Department of Clinical Sciences and Community Health Università degli Studi di Milano, 20133 Milan, Italy

^26^ Division of Cancer Epidemiology, German Cancer Research Center (DKFZ), Heidelberg, Germany

^27^ Research Group Genomic Epidemiology, German Cancer Research Center (DKFZ), Heidelberg, Germany

^28^ Department of Epidemiology and Biostatistics, School of Public Health, Imperial College London, London, United Kingdom

^29^ Department of Nutrition, Bjørknes University College, Oslo, Norway

^30^ Department of Endocrinology, Morbid Obesity and Preventive Medicine, Oslo University Hospital, Oslo, Norway

^31^ Department of Epidemiology, German Institute of Human Nutrition Potsdam-Rehbruecke, Nuthetal, Germany

^32^ Department of Molecular Epidemiology, German Institute of Human Nutrition Potsdam-Rehbruecke, Nuthetal, Germany

^33^ University of Potsdam, Institute of Nutritional Sciences, Nuthetal, Germany

^34^ Cancer Epidemiology Unit, Nuffield Department of Population Health, University of Oxford, Oxford, United Kingdom

^35^ Julius Center for Health Sciences and Primary Care, University Medical Center Utrecht, Utrecht, The Netherlands

^36^ MRC-PHE Centre for Environment and Health, Department of Epidemiology and Biostatistics, Imperial College London, London, UK

**Supplementary materials**

**Supplementary Table 1.** Spearman correlations and p values between immune markers

|  |  | **sCD23** | **sCD27** | **sCD30** |
| --- | --- | --- | --- | --- |
| **Controls** | **sCD27** | 0.14, 0.002 |  |  |
|  | **sCD30** | 0.29, <0.0001 | 0.32, <0.0001 |  |
|  | **CXCL13** | 0.17, <0.0001 | 0.14, 0.002 | 0.20, <0.0001 |
| **BCL** | **sCD27** | 0.40, <0.0001 |  |  |
|  | **sCD30** | 0.40, <0.0001 | 0.51, <0.0001 |  |
|  | **CXCL13** | 0.25, <0.0001 | 0.28, <0.0001 | 0.43, <0.0001 |
| **CLL** | **sCD27** | 0.50, <0.0001 |  |  |
|  | **sCD30** | 0.36, <0.0001 | 0.63, <0.0001 |  |
|  | **CXCL13** | 0.25, <0.0001 | 0.29, <0.0001 | 0.39, <0.0001 |
| **FL** | **sCD27** | 0.45, <0.0001 |  |  |
|  | **sCD30** | 0.60, <0.0001 | 0.62, <0.0001 |  |
|  | **CXCL13** | 0.40, <0.0001 | 0.42, <0.0001 | 0.62, <0.0001 |
| **DLBCL** | **sCD27** | 0.36, <0.0001 |  |  |
|  | **sCD30** | 0.36, <0.0001 | 0.52, <0.0001 |  |
|  | **CXCL13** | 0.33, <0.0001 | 0.39, <0.0001 | 0.53, <0.0001 |

B-cell lymphoma (BCL), diffuse large B-cell lymphoma (DLBCL), follicular lymphoma (FL), chronic lymphocytic leukemia (CLL)

**Supplementary Table 2.** Serum level of immune markers stratified by gender and country

|  | **Gender** | | | **Country** | | | | | | |
| --- | --- | --- | --- | --- | --- | --- | --- | --- | --- | --- |
|  | **Male** | **Female** | *p* | **Italy** | **Spain** | **UK** | **Netherlands** | **Greece** | **Germany** | *p* |
| **sCD23** | 7.93 (0.64) | 7.93 (0.54) | 0.89 | 7.86 (0.5) | 7.92 (0.62) | 7.97 (0.59) | 7.92 (0.55) | 8.19 (0.59) | 7.88 (0.63) | 0.23 |
| **sCD27** | 3.55 (0.37) | 3.51 (0.35) | 0.14 | 3.52 (0.35) | 3.59 (0.37) | 3.52 (0.36) | 3.49 (0.37) | 3.66 (0.39) | 3.40 (0.32) | 0.09 |
| **sCD30** | 2.71 (0.4) | 2.80 (0.5) | **0.001** | 2.69 (0.4) | 2.79 (0.5) | 2.83 (0.47) | 2.78 (0.51) | 2.94 (0.37) | 2.57 (0.36) | 0.26 |
| **CXCL13** | 3.17 (0.63) | 3.27 (0.78) | **0.02** | 3.09 (0.77) | 2.99 (0.78) | 3.45 (0.63) | 3.27 (0.72) | 3.45 (0.55) | 3.18 (0.67) | **0.002** |

Mean (SD); unit of measurement is pg/mL for sCD23 and CXCL13, ng/ml for sCD30, and U/ml for sCD27

**Supplementary Table 3.** Univariate and multivariable conditional regression analyses: odds ratio (OR) and 95% confidence interval for immune marker measurements (continuous variables) and B-cell lymphoma and subtypes

|  | **BCL** | **CLL** | **FL** | **DLBCL** |
| --- | --- | --- | --- | --- |
| **sCD23,** pg/mL | N=516 | N=210 | N=132 | N=174 |
| *Uni-OR (95%CI)* | 6.7 (4.6-9.7) | 11.0 (5.7-21.2) | 4.8 (2.3-10.0) | 4.5 (2.4-8.5) |
| *p* | <0.0001 | <0.0001 | <0.0001 | <0.0001 |
| *Multi-OR (95%CI)* | 7.7 (5.14-11.5) | 14.2 (6.6-30.7) | 5.52 (2.5-12.0) | 5.14 (2.4-10.9) |
| *p* | <0.0001 | <0.0001 | <0.0001 | <0.0001 |
| **sCD30,** ng/ml | N=516 | N=210 | N=132 | N=174 |
| *Uni-OR (95%CI)* | 3.5 (2.4-5.2) | 2.9 (1.6-5.2) | 3.5 (1.7-7.0) | 4.7 (2.3-9.6) |
| *p* | <0.0001 | 0.0003 | 0.001 | <0.0001 |
| *Multi-OR (95%CI)* | 3.65 (2.48-5.4) | 3.25 (1.74-6.1) | 4.08 (1.89-8.8) | 5.15 (2.4-11.3) |
| *p* | <0.0001 | 0.0002 | 0.0004 | <0.0001 |
| **sCD27,** U/ml | N=511 | N=207 | N=131 | N=173 |
| *Uni-OR (95%CI)* | 7.8 (4.2-14.2) | 9.6 (3.8-24.6) | 7.1 (2.1-23.9) | 6.2 (2.1-17.8) |
| *p* | <0.0001 | <0.0001 | 0.002 | 0.001 |
| *Multi-OR (95%CI)* | 7.9 (4.3-14.63) | 12.97 (4.52-37.2) | 8.02 (2.1-30.6) | 6.6 (2.08-21.0) |
| *p* | <0.0001 | <0.0001 | 0.002 | 0.002 |
| **CXCL13,** pg/mL | N=506 | N=204 | N=128 | N=174 |
| *Uni-OR (95%CI)* | 2.1 (1.65-2.6) | 1.5 (1.05-2.15) | 2.4 (1.56-3.79) | 2.6 (1.7-3.9) |
| *p* | <0.0001 | 0.03 | <0.0001 | <0.0001 |
| *Multi-OR (95%CI)* | 2.1 (1.67-2.6) | 1.5 (1.05-2.2) | 2.5 (1.58-3.94) | 2.8 (1.8-4.4) |
| *p* | <0.0001 | 0.03 | <0.0001 | <0.0001 |
| **Full model (combined)** | N=503 | N=203 | N=127 | N=173 |
| sCD23 | 2.88 (2.25-3.70) | 4.38 (2.76-6.94) | 2.40 (1.40-4.15) | 1.74 (1.06-2.86) |
| sCD30 | 0.95 (0.73-1.23) | 0.83 (0.49-1.40) | 0.86 (0.50-1.48) | 1.34 (0.86-2.08) |
| sCD27 | 1.45 (1.06-1.98) | 1.60 (0.84-3.06) | 1.53 (0.78-3.02) | 1.34 (0.79-2.27) |
| CXCL13 | 1.47 (1.20-1.82) | 1.03 (0.66-1.56) | 1.82 (1.21-2.73) | 1.66 (1.16-2.38) |

Models are based on one unit increments; B-cell lymphoma (BCL), diffuse large B-cell lymphoma (DLBCL), follicular lymphoma (FL), chronic lymphocytic leukemia (CLL); multivariable models adjusted for BMI, education, smoking, alcohol intake, and physical activity

**Supplementary Table 4.** Odds ratio (OR) and 95% confidence interval (CI) for individual immune marker (categorical variable) and B-cell lymphoma and histological subtypes for cases diagnosed after more than 9 years of follow-up

|  | **BCL** | | **CLL** | | **FL** | | **DLBCL** | |
| --- | --- | --- | --- | --- | --- | --- | --- | --- |
| **sCD23** | **N** | **OR (95% CI)** | **N** | **OR (95% CI)** | **N** | **OR (95% CI)** | **N** | **OR (95% CI)** |
| *Q1* | 39/131 | Ref. | 11/131 | Ref. | 14/131 | Ref. | 14/131 | Ref. |
| *Q2* | 44/131 | 1.09 (0.70-1.70) | 11/131 | 0.91 (0.38-2.15) | 16/131 | 1.27 (0.60-2.71) | 17/131 | 1.24 (0.59-2.58) |
| *Q3* | 66/132 | 1.61 (1.06-2.45) | 21/132 | 1.82 (0.84-3.94) | 19/132 | 1.52 (0.77-3.22) | 26/132 | 2.12 (1.06-4.22) |
| *Q4* | 109/131 | 2.17 (1.47-3.21) | 52/131 | 3.70 (1.85-7.39) | 11/131 | 1.08 (0.46-2.53) | 46/131 | 3.21 (1.68-6.12) |
| *P* |  | **<0.0001** |  | **<0.0001** |  | 0.69 |  | **<0.0001** |
| **sCD30** |  |  |  |  |  |  |  |  |
| *Q1* | 56/130 | **Ref.** | 18/130 | Ref. | 21/130 | Ref. | 17/130 | Ref. |
| *Q2* | 55/132 | 1.00 (0.67-1.48) | 17/132 | 0.90 (0.45-1.82) | 9/132 | 0.43 (0.19-0.99) | 29/132 | 1.72 (0.90-3.27) |
| *Q3* | 67/133 | 1.16 (0.79-1.70) | 24/133 | 1.12 (0.57-2.19) | 17/133 | 1.08 (0.53-2.20) | 26/133 | 1.68 (0.87-3.26) |
| *Q4* | 80/130 | 1.36 (0.93-1.99) | 36/130 | 1.64 (0.87-3.10) | 13/130 | 0.74 (0.35-1.60) | 31/130 | 2.15 (1.12-4.15) |
| *P* |  | 0.07 |  | 0.06 |  | 0.88 |  | **0.04** |
| **sCD27** |  |  |  |  |  |  |  |  |
| *Q1* | 50/134 | Ref. | 17/134 | Ref. | 12/134 | Ref. | 21/134 | Ref. |
| *Q2* | 62/129 | 1.35 (0.90-2.05) | 23/129 | 1.61 (0.81-3.19) | 18/129 | 1.77 (0.76-4.12) | 21/129 | 1.33 (0.67-2.66) |
| *Q3* | 63/132 | 1.44 (0.92-2.25) | 27/132 | 2.09 (1.01-4.32) | 13/132 | 1.45(0.54-3.89) | 23/132 | 1.51 (0.73-3.10) |
| *Q4* | 83/130 | 2.06 (1.30-3.28) | 28/130 | 2.72 (1.25-5.92) | 17/130 | 2.19 (0.76-6.33) | 38/130 | 3.07 (1.47-6.43) |
| *P* |  | **0.003** |  | **0.01** |  | 0.21 |  | **0.002** |
| **CXCL13** |  |  |  |  |  |  |  |  |
| *Q1* | 56/135 | **Ref.** | 26/135 | Ref. | 15/135 | Ref. | 15/135 | Ref. |
| *Q2* | 51/130 | 1.04 (0.69-1.55) | 19/130 | 0.80 (0.42-1.52) | 17/130 | 1.21 (0.57-2.56) | 15/130 | 1.19 (0.56-2.53) |
| *Q3* | 69/130 | 1.29 (0.88-1.89) | 24/130 | 0.93 (0.50-1.74) | 10/130 | 0.82 (0.35-1.92) | 35/130 | 2.46 (1.29-4.70) |
| *Q4* | 82/130 | 1.48 (1.01-2.15) | 26/130 | 0.99 (0.53-1.85) | 18/130 | 1.61 (0.74-3.47) | 38/130 | 2.87 (1.49-5.55) |
| *P* |  | **0.02** |  | 0.90 |  | 0.37 |  | **0.0002** |

B-cell lymphoma (BCL), diffuse large B-cell lymphoma (DLBCL), follicular lymphoma (FL), chronic lymphocytic leukemia (CLL); adjusted for BMI, education, smoking, alcohol intake, physical activity, age, sex, country, and plate; to see the immune marker levels associated with each quartile see Table 2.

**Supplementary Table 5.** Cross-validated AUC of ROC curves for the prediction of future lymphoma diagnosis by individual and combined markers

|  | **10-fold cross-validated AUC** | | |
| --- | --- | --- | --- |
|  | **CLL** | **FL** | **DLBCL** |
| sCD23 | 0.796 (0.757-0.835) | 0.605 (0.549-0.660) | 0.625 (0.577-0.673) |
| sCD30 | 0.582 (0.536-0.629) | 0.546 (0.490-0.602) | 0.582 (0.533-0.632) |
| sCD27 | 0.585 (0.538-0.631) | 0.534 (0.480-0.587) | 0.554 (0.504-0.604) |
| CXCL13 | 0.517 (0.471-0.563) | 0.588 (0.531-0.645) | 0.627 (0.578-0.676) |
| sCD23 + sCD30 | 0.792 (0.752-0.832) | 0.602 (0.546-0.657) | 0.630 (0.582-0.678) |
| sCD23 + sCD27 | 0.763 (0.753-0.833) | 0.611 (0.555-0.667) | 0.628 (0.580-0.676) |
| sCD23 + CXCL13 | 0.789 (0.748-0.829) | 0.638 (0.585-0.691) | 0.663 (0.615-0.711) |
| sCD30 + sCD27 | 0.601 (0.555-0.648) | 0.552 (0.497-0.606) | 0.582 (0.532-0.632) |
| sCD30 + CXCL13 | 0.582 (0.535-0.629) | 0.596 (0.539-0.653) | 0.631 (0.582-0.680) |
| sCD27 + CXCL13 | 0.582 (0.536-0.629) | 0.604 (0.549-0.660) | 0.627 (0.577-0.676) |
| sCD23 + sCD30 + sCD27 | 0.791 (0.751-0.831) | 0.611 (0.554-0.667) | 0.626 (0.578-0.674) |
| sCD23 + sCD30 + CXCL13 | 0.790 (0.750-0.830) | 0.633 (0.580-0.687) | 0.659 (0.611-0.707) |
| sCD23 + CXCL13 + sCD27 | 0.790 (0.750-0.830) | 0.638 (0.584-0.692) | 0.657 (0.609-0.706) |
| CXCL13 + sCD30 + sCD27 | 0.596 (0.549-0.642) | 0.603 (0.547-0.658) | 0.630 (0.580-0.679) |
| sCD23 + sCD30 + sCD27 + CXCL13 | 0.788 (0.748-0.828) | 0.634 (0.580-0.688) | 0.655 (0.607-0.704) |

Diffuse large B-cell lymphoma (DLBCL), follicular lymphoma (FL), chronic lymphocytic leukemia (CLL)

**Supplementary Table 6.** AUC of ROC curves for the prediction of future CLL diagnosis by sCD23 stratified by gender and age at recruitment

|  |  | **10-fold cross-validated AUC** |
| --- | --- | --- |
| Sex | Male | 0.853 (0.807-0.900) |
|  | Female | 0.739 (0.678-0.800) |
| Age categories | <50 | 0.667 (0.582-0.752) |
|  | 50-60 | 0.830 (0.770-0.890) |
|  | >60 | 0.844 (0.788-0.901) |
| Age categories in male | <50 | 0.595 (0.491-0.699) |
|  | 50-60 | 0.868 (0.801-0.936) |
|  | >60 | 0.882 (0.822-0.942) |
| Age categories in female | <50 | 0.594 (0.476-0.713) |
|  | 50-60 | 0.790 (0.705-0.875) |
|  | >60 | 0.777 (0.684-0.871) |

**Supplementary Table 7.** Test accuracy parameter for different cut-off values of sCD23 for CLL stratified by gender

|  | **sCD23 cut-off levels, pg/mL** | | | | | | |
| --- | --- | --- | --- | --- | --- | --- | --- |
|  | 30th | 40th | 50th | 60th | 70th | 80th | 90th |
|  | ≥ 1832.3 | ≥ 2049.9 | ≥ 2255.7 | ≥ 2453.3 | ≥ 2717.5 | ≥ 3018.8 | ≥ 3608.3 |
| Sensitivity | 0.92 | 0.88 | 0.83 | 0.79 | 0.73 | 0.65 | 0.55 |
| Specificity | 0.30 | 0.40 | 0.50 | 0.60 | 0.70 | 0.80 | 0.90 |
| PPV | 0.35 | 0.37 | 0.40 | 0.44 | 0.50 | 0.57 | 0.69 |
| NPV | 0.91 | 0.89 | 0.88 | 0.88 | 0.87 | 0.85 | 0.83 |
|  |  |  | **Male** |  |  |  |  |
| Sensitivity | 0.98 | 0.93 | 0.88 | 0.85 | 0.80 | 0.71 | 0.65 |
| Specificity | 0.35 | 0.46 | 0.58 | 0.67 | 0.76 | 0.82 | 0.91 |
| PPV | 0.40 | 0.44 | 0.48 | 0.54 | 0.59 | 0.64 | 0.76 |
| NPV | 0.98 | 0.94 | 0.91 | 0.91 | 0.89 | 0.86 | 0.85 |
|  |  |  | **Female** |  |  |  |  |
| Sensitivity | 0.88 | 0.84 | 0.80 | 0.74 | 0.67 | 0.60 | 0.46 |
| Specificity | 0.26 | 0.36 | 0.44 | 0.55 | 0.66 | 0.78 | 0.90 |
| PPV | 0.30 | 0.32 | 0.34 | 0.38 | 0.42 | 0.50 | 0.62 |
| NPV | 0.85 | 0.86 | 0.85 | 0.85 | 0.85 | 0.84 | 0.82 |

Cut off values are based on deciles (10th, 20th, …., 90th) of sCD23 levels among control subjects. Results for cut-off values of first two deciles were not shown. Positive predictive value (PPV); negative predictive value (NPV).

**Supplementary Table 8.** Test accuracy parameter for different cut-off values of sCD23 and CXCL13 for DLBCL and FL stratified by gender

|  | **FL** | | | | | **DLBCL** | | | | |
| --- | --- | --- | --- | --- | --- | --- | --- | --- | --- | --- |
|  | **50th** | **60th** | **70th** | **80th** | **90th** | **50th** | **60th** | **70th** | **80th** | **90th** |
| **sCD23 cut-off levels** | **≥ 2255.7** | **≥ 2453.3** | **≥ 2717.5** | **≥ 3018.8** | **≥ 3608.3** | **≥ 2255.7** | **≥ 2453.3** | **≥ 2717.5** | **≥ 3018.8** | **≥ 3608.3** |
| Sensitivity | 0.67 | 0.63 | 0.49 | 0.37 | 0.29 | 0.66 | 0.62 | 0.51 | 0.37 | 0.22 |
| Specificity | 0.50 | 0.60 | 0.70 | 0.80 | 0.90 | 0.50 | 0.60 | 0.70 | 0.80 | 0.90 |
| PPV | 0.25 | 0.28 | 0.29 | 0.32 | 0.42 | 0.30 | 0.34 | 0.36 | 0.38 | 0.42 |
| NPV | 0.86 | 0.87 | 0.84 | 0.84 | 0.83 | 0.82 | 0.83 | 0.81 | 0.79 | 0.78 |
|  |  |  | **Male** |  |  |  |  |  |  |  |
| Sensitivity | 0.58 | 0.57 | 0.40 | 0.31 | 0.20 | 0.58 | 0.54 | 0.44 | 0.33 | 0.19 |
| Specificity | 0.58 | 0.67 | 0.76 | 0.82 | 0.91 | 0.58 | 0.67 | 0.76 | 0.82 | 0.91 |
| PPV | 0.22 | 0.26 | 0.25 | 0.26 | 0.31 | 0.31 | 0.35 | 0.37 | 0.38 | 0.41 |
| NPV | 0.87 | 0.88 | 0.86 | 0.85 | 0.85 | 0.81 | 0.82 | 0.81 | 0.79 | 0.78 |
|  |  |  | **Female** |  |  |  |  |  |  |  |
| Sensitivity | 0.71 | 0.67 | 0.53 | 0.40 | 0.33 | 0.72 | 0.67 | 0.56 | 0.40 | 0.24 |
| Specificity | 0.44 | 0.55 | 0.66 | 0.78 | 0.90 | 0.44 | 0.55 | 0.66 | 0.78 | 0.90 |
| PPV | 0.27 | 0.30 | 0.31 | 0.35 | 0.48 | 0.30 | 0.33 | 0.36 | 0.38 | 0.43 |
| NPV | 0.84 | 0.85 | 0.83 | 0.82 | 0.83 | 0.82 | 0.83 | 0.82 | 0.80 | 0.78 |
| **CXCL13 cut-off levels** | **≥ 22.0** | **≥ 25.4** | **≥ 29.5** | **≥ 34.4** | **≥ 43.1** | **≥ 22.0** | **≥ 25.4** | **≥ 29.5** | **≥ 34.4** | **≥ 43.1** |
| Sensitivity | 0.64 | 0.61 | 0.49 | 0.42 | 0.27 | 0.72 | 0.66 | 0.49 | 0.37 | 0.26 |
| Specificity | 0.50 | 0.60 | 0.70 | 0.80 | 0.90 | 0.50 | 0.60 | 0.70 | 0.80 | 0.90 |
| PPV | 0.24 | 0.27 | 0.29 | 0.34 | 0.40 | 0.33 | 0.35 | 0.36 | 0.38 | 0.46 |
| NPV | 0.85 | 0.86 | 0.85 | 0.85 | 0.83 | 0.84 | 0.84 | 0.81 | 0.79 | 0.78 |
|  |  |  | **Male** |  |  |  |  |  |  |  |
| Sensitivity | 0.52 | 0.50 | 0.41 | 0.32 | 0.16 | 0.69 | 0.58 | 0.40 | 0.32 | 0.19 |
| Specificity | 0.51 | 0.62 | 0.73 | 0.83 | 0.94 | 0.51 | 0.62 | 0.73 | 0.83 | 0.94 |
| PPV | 0.18 | 0.21 | 0.23 | 0.28 | 0.33 | 0.32 | 0.33 | 0.33 | 0.38 | 0.50 |
| NPV | 0.84 | 0.86 | 0.86 | 0.86 | 0.85 | 0.84 | 0.82 | 0.79 | 0.79 | 0.78 |
|  |  |  | **Female** |  |  |  |  |  |  |  |
| Sensitivity | 0.70 | 0.67 | 0.54 | 0.48 | 0.33 | 0.75 | 0.71 | 0.56 | 0.40 | 0.30 |
| Specificity | 0.50 | 0.59 | 0.68 | 0.78 | 0.87 | 0.50 | 0.59 | 0.68 | 0.78 | 0.87 |
| PPV | 0.28 | 0.31 | 0.32 | 0.37 | 0.42 | 0.33 | 0.37 | 0.38 | 0.38 | 0.45 |
| NPV | 0.86 | 0.86 | 0.84 | 0.84 | 0.82 | 0.85 | 0.86 | 0.82 | 0.79 | 0.79 |

Cut off values are based on deciles (10th, 20th, …., 90th) of the marker level in control subjects. Results for cut-off values of first four deciles were not shown. Positive predictive value (PPV); negative predictive value (NPV)

**Supplementary Table 9.** Association between risk factors and level of B-cell activation markers (Model M) and BCL subtype (Model X)

| **sCD23** | **Model M*** | | | | | | **Model X** | | | | | |
| --- | --- | --- | --- | --- | --- | --- | --- | --- | --- | --- | --- | --- |
|  | CLL |  | FL |  | DLBCL |  | CLL |  | FL |  | DLBCL |  |
|  | Estimate (SE) | P | Estimate (SE) | P | Estimate (SE) | P | Estimate (SE) | P | Estimate (SE) | P | Estimate (SE) | P |
| Height | 0.04 (0.06) | 0.55 | -0.04 (0.05) | 0.43 | 0.03 (0.04) | 0.51 | 0.19 (0.13) | 0.16 | 0.06 (0.15) | 0.69 | **0.32 (0.14)** | **0.03** |
| BMI | **0.10 (0.06)** | **0.08** | 0.06 (0.05) | 0.21 | **0.08 (0.04)** | **0.04** | **0.27 (0.13)** | **0.04** | 0.05 (0.15) | 0.74 | -0.17 (0.15) | 0.26 |
| Smoking | -0.02 (0.05) | 0.72 | -0.003 (0.04) | 0.94 | -0.06 (0.04) | 0.10 | 0.07 (0.12) | 0.58 | 0.07 (0.14) | 0.63 | -0.20 (0.14) | 0.14 |
| Activity | 0.04 (0.05) | 0.45 | -0.05 (0.04) | 0.21 | -0.02 (0.04) | 0.60 | 0.15 (0.12) | 0.22 | -0.03 (0.14) | 0.85 | -0.05 (0.13) | 0.70 |
| Alcohol | **-0.12 (0.06)** | **0.04** | 0.001 (0.05) | 0.98 | -0.04 (0.04) | 0.31 | -0.15 (0.13) | 0.24 | 0.04 (0.16) | 0.80 | 0.05 (0.15) | 0.72 |
| Education | -0.03 (0.05) | 0.60 | 0.01 (0.04) | 0.81 | -0.02 (0.03) | 0.49 | -0.13 (0.11) | 0.22 | 0.03 (0.12) | 0.81 | **-0.37 (0.12)** | **0.002** |
| **CXCL13** |  |  |  |  |  |  |  |  |  |  |  |  |
| Height |  |  | 0.04 (0.07) | 0.57 | 0.07 (0.07) | 0.33 |  |  |  |  |  |  |
| BMI |  |  | 0.07 (0.07) | 0.32 | **0.15 (0.07)** | **0.03** |  |  |  |  |  |  |
| Smoking |  |  | 0.06 (0.07) | 0.38 | -0.03 (0.06) | 0.67 |  |  |  |  |  |  |
| Activity |  |  | **-0.12 (0.07)** | **0.08** | **-0.16 (0.06)** | **0.01** |  |  |  |  |  |  |
| Alcohol |  |  | 0.11 (0.08) | 0.13 | 0.01 (0.07) | 0.85 |  |  |  |  |  |  |
| Education |  |  | 0.04 (0.06) | 0.49 | 0.02 (0.06) | 0.65 |  |  |  |  |  |  |

B-cell lymphoma (BCL), diffuse large B-cell lymphoma (DLBCL), follicular lymphoma (FL), chronic lymphocytic leukemia (CLL); * Examination was performed among case and controls together; smoking: non-smoker at recruitment (0) vs. smoker (1); alcohol intake: non-drinker (0) vs. drinker (1), activity: first 3 categories (0) vs. active(1); education: first 3 categories (0) vs. secondary school/ university/college (1); BMI: <30 (0) vs. ≥30 (1); height: <country median (0) vs. ≥country median (1); **Model M** (Linear regression): Immune marker ~ Sex + Country + Age + Height + BMI + Smoking + Activity + Alcohol intake + Education; **Model X** (Logistic regression): Case-control status ~ Sex + Country + Age + BMI + Height +Smoking + Activity + Alcohol intake + Education.

**Supplementary Table 10.** Missing rate of covariates and immune markers

| Variable | % out of 1042 |
| --- | --- |
| Education | 6.9 |
| Alcohol intake | 0.38 |
| Physical activity | 2.7 |
| Smoking status | 2.02 |
| sCD23 | 6.33 (>highest detection limit) |
| sCD30 | 0.77 (<lowest detection limit) |
| sCD27 | 0.48 (n=5 not enough sample)* |
| CXCL13 | 19.1 (<lowest detection limit (18.9%) or > highest detection limit (0.2%)) (n=11 not enough sample*) |

* not imputed

**Supplementary Figure 1.** Correlation between serum level of immune markers and recruitment age by case-control status
